# The association between feelings of loneliness and the number of social relationships in depression: a cross-sectional study of German adults

**DOI:** 10.1186/s12888-026-07915-3

**Published:** 2026-02-28

**Authors:** Valeria Koppert, Andreas Czaplicki, Ulrich Hegerl

**Affiliations:** 1 Depression Research Centre of the German Foundation for Depression and Suicide Prevention, Department of Psychiatry, Psychosomatic Medicine and Psychotherapy, Goethe University Frankfurt, University Hospital, Frankfurt am Main, Germany; 2 German Foundation for Depression and Suicide Prevention, Leipzig, Germany; 3 Goethe Research Professorship, Department of Psychiatry, Psychosomatic Medicine and Psychotherapy, Goethe University Frankfurt, University Hospital, Frankfurt am Main, Germany

**Keywords:** Depression, Loneliness, Social relationships, Social contacts, Social isolation

## Abstract

**Background:**

Depression is a common psychiatric disorder that is associated with both feelings of loneliness and withdrawal from social relationships. However, clinical experience suggests that feelings of loneliness are an integral part of depressive symptomatology and can occur even when a patient has many social relationships. We addressed the following three research questions: 1. How are feelings of loneliness and the number of social relationships associated in people with depression compared with the nondepressed population? 2. Do people with depression have more feelings of loneliness compared with the nondepressed population? 3. Do people with depression have fewer social relationships compared with the nondepressed population?

**Methods:**

Cross-sectional data from the German Depression Barometer 2023, a representative survey of the German adult population (N=5196), were analysed. Feelings of loneliness and the number of social relationships were assessed via self-reports.

**Results:**

Feelings of loneliness and the number of social relationships are negatively correlated both in people with a lifetime diagnosis of depression (n=1221, Spearman’s ρ = -.234, p <.001, 95% CI [-.288; -.179]) and in people without a depression diagnosis (n=2821, Spearman’s ρ = -.132, p <.001, 95% CI [-.169; -.094]). The strength of the correlation is significantly greater for people with a lifetime diagnosis of depression (Z=2.99, p <.02). Increased feelings of loneliness are found in people with a lifetime diagnosis of depression (*M*=6.53) compared with the nondepressed German population (*M*=4.30). The difference between the two groups is significant, t(4040) = 19.08 , p <.001, d = 0.654. Additionally, fewer social relationships are found in people with a lifetime diagnosis of depression (39.2% with 0-4 social contacts on a normal day) than in the nondepressed German population (20.5% with 0-4 social contacts on a normal day). The difference between the two groups is again significant, U = 1247054,50, Z = -14.44, p <.001, r = 0.227.

**Conclusions:**

Contrary to our expectations, a stronger inverse correlation between feelings of loneliness and the number of social relationships was observed in people with depression than in the nondepressed population. Furthermore, we replicated findings of increased feelings of loneliness and a reduced number of social relationships in people with a lifetime diagnosis of depression. This has possible implications for treatment decisions and intervention programs, namely, that both feelings of loneliness and social relationships should be addressed.

**Clinical trial number:**

Not applicable.

**Supplementary Information:**

The online version contains supplementary material available at 10.1186/s12888-026-07915-3.

## Background

Depression is a serious and common psychiatric disorder. The 12-month prevalence of unipolar depressive disorders in adults in Germany is 8.2% [1], and the lifetime prevalence is 19% [2]. The relevance for society is high, as mental disorders were responsible for 1,898.5 age-standardised disability-adjusted life years (DALYs) per 100,000 people in Germany in 2019 [3]. Depressive disorders account for the highest proportion of DALYs due to psychiatric disorders, at 37.3% [3].

Having a depressive disorder is correlated with feelings of loneliness with a moderate effect size (*r* =.50, *p* <.01) [4, 5]. Feelings of loneliness are associated with symptoms of depression [6] and are used in scales to measure depression (e.g. CES-D item 14: “I felt lonely”) [7]. Conversely, depression disorder has been found to lead to more feelings of loneliness [8], and feelings of loneliness have been found to predict poorer depression outcome [9–11].

Feelings of loneliness are defined as the subjective perception of a mismatch between actual and desired social relationships [12, 13]. Feelings of loneliness represent a functional aspect in conceptualising and measuring people’s social relationships [14]. A considerable proportion of the population is affected by feelings of loneliness. In the EU, 6% of people report frequent feelings of loneliness [15]; in Germany, one in ten Germans feels lonely often or very often [16]. Loneliness has been found to have a negative effect on people’s well-being, physical health and life expectancy [10, 17–19].

A depressive disorder can lead affected patients to have fewer social contacts through social withdrawal [20, 21]. People with a depressive disorder show less interest in social interactions [22], which puts them at high risk of losing their social relationships or failing to make new ones. Overall, depression has been found to be correlated with fewer social relationships (*r* =.21, *p* <.001) [23].

Social isolation is an objective construct used to assess the extent to which people miss social relationships [24]. The term is often used imprecisely in the literature, making it difficult to compare results [14, 25]. Methodological analyses suggest that social isolation represents the number (quantity) and quality of social relationships [26] and captures the structural aspect of measuring social relationships [14].

The distinction between subjective perceptions of social relationships, feelings of loneliness, and the objective structure of social relationships is important, as each has a unique and significant relationship with mental and physical health [27], and both are relevant and distinct risk factors for mortality [19].

When the different social relationship domains of people are depicted on a conceptual map, feelings of loneliness and the number of social relationships are not contiguous [25]. The two domains represent the highest level of the respective subjective and objective measure of social relationships. It is important to understand the association between the two social relationship domains, particularly in people with depression, to provide reliable evidence for intervention development and evaluation [25]. In this study, we aim to examine the association between feelings of loneliness and the number of social relationships.

In healthy people, there is a negative correlation between the two measures: the fewer social contacts people have, the more feelings of loneliness they experience (*r* = −.31, *p* <.001) [28]. In patients with persistent depressive disorder (PDD), no correlation was found between feelings of loneliness and the number of social relationships, suggesting a different association of both social relationship domains in PDD patients than in healthy individuals [29]. Other than this finding, the association between different social relationship domains has rarely been researched in people with mental disorders [14].

PDD is a DSM-5 diagnosis that is part of the depressive disorders and includes dysthymia and chronic depression [30]. The focus of our study is episodic depression. As episodic depression and PDD are both depressive disorders and therefore share symptoms such as a “depressed mood” [31], we will examine whether there is a similar association between the subjective and objective social relationship domains in episodic depression patients as there is in PDD patients.

Because of the episodic nature of depression, we additionally assess the current state of depression. This is important because people with depression may be severely affected by acute symptoms, or they may be asymptomatic because they have recovered from their last episode of depression [32].

The resulting main research question is as follows:

1. How are feelings of loneliness and the number of social relationships associated in people with depression compared with the nondepressed population?

In addition, we aim to study the state dependency of this main question by comparing three subgroups of the overall depression group (acute depressive phase, residual symptomatic phase and not symptomatic phase of episodic depressive disorder):

Presumed the association is different, does the difference vary with respect to the current state of lifetime episodic depression?

People with PDD are known to feel more lonely than healthy people [29], and people with episodic depression are known to withdraw socially [20]. Our study aims to provide evidence related to people with episodic depression. We aim to confirm the simultaneous existence of two associations: first, depression and feelings of loneliness and second, depression and the number of social relationships in the German population and to compare the effect sizes with those of healthy people. In doing so, we aim to update and extend previous findings on loneliness in representative German population data analysed by Beutel et al. [10].

The secondary research questions are as follows:

2. Do people with depression have more feelings of loneliness compared with the nondepressed population?
3. Do people with depression have fewer social relationships compared with the nondepressed population?

## Methods

### Aim of the study

The study was conducted to answer the introduced research questions. The following hypotheses were tested:

1. In people with depression there is a different association between feelings of loneliness and the number of social relationships than in people without depression.
  - *For people with depression* (lifetime diagnosis), we assume no correlation between feelings of loneliness and the number of social relationships [29].
  - *For people without depression*, we assume a correlation between feelings of loneliness and the number of social relationships.
  - The extent to which the correlations differ may depend on the current state of depression. When correlations in three *subgroups of depression* (acute depressive phase – residual symptomatic phase – not symptomatic phase) are researched, the difference described in 1a/1b is thought to be most prominent among people experiencing an acute depressive phase.
2. People with depression feel lonelier than people without depression.
  - This finding is expected to depend on the current state of depression. When the three subgroups of depression are studied, it is assumed that the difference in loneliness will be greatest for people experiencing an acute depressive phase.
3. People with depression have fewer social relationships than people without depression.
  - This finding is expected to depend on the current state of depression. When the three subgroups of depression are studied, it is assumed that the difference in the number of social relationships will be greatest for people experiencing an acute depressive phase.

### Study design and setting

We used a cross-sectional study design. Data were collected through the German Depression Barometer (Deutschland Barometer Depression), an annual national survey of people aged 18–69 years in Germany conducted by the German Foundation for Depression and Suicide Prevention, which has been supported by the “Deutsche Bahn Stiftung”, since 2017 [33]. Data were collected from 28th August to 8th September 2023. The survey was carried out by Bilendi, a certified survey company. It used a pool of people who are generally willing to participate in anonymous surveys. The participants in our survey were selected based on sex, age and region of residence. This selection resulted in a representative sample of the German population based on current population data published by the German Federal Statistical Office.

To obtain the representative sample, a multiple stratified quota sample with 70 quota cells was used (the foundation of the specifications in the quota cells were the current population data of the Federal Statistical Office). It was based on the mentioned interleaved characteristics: sex, age, and federal state. The weighting of the data accounts for minor deviations between the total sample size and the sum of the sample sizes of the subgroups.

People were contacted via e-mail and were not given any information about the subject of the survey. This minimised the risk of self-selection. 15.6% of people contacted responded to the invitation letter and started the survey, leading to a final completion rate of 12.2% due to dropouts for various reasons (e.g., cancelled interviews, overrepresented quota cells). The data were collected anonymously in an online survey. No increased drop-out rate was found after people were told the topic of the survey (depression). The participants were asked about their own mental health status and their knowledge and opinions about depression. In 2023, questions about feelings of loneliness and the number of daily social contacts were added to the standard items about depression.

The sampling resulted in *N* = 5196 completed questionnaires. The respondents were aged 18–69 years.

### Variables and instruments

The relevant characteristics of the participants for answering the research questions are their mental health status in terms of depression, their level of feelings of loneliness and the number of their social relationships.

#### Depression groups

People with depression are participants who reported having or ever having been diagnosed with depression by a doctor (lifetime diagnosis). The participants received the following item (in German language): ‘Have you ever been in personal contact with depression?’ Different answer options were presented, with multiple choices possible. People who answered ‘Yes, I have been diagnosed with depression before.’ were included in the overall depression group.

To divide the depression group into three depression subgroups the overall depression group received the following item: ‘How severely are you currently affected by depression?’. The answer options were ‘I am currently in a depressive phase.’, ‘I currently have some residual symptoms, but I am no longer in an acute depressive phase.’, ‘I currently have no symptoms of depression.’ and ‘I don’t know’. In this way, the current phase of depression (acute depressive phase – residual symptomatic phase – not symptomatic phase) was distinguished for subsequent analyses.

Research suggests that the prevalence is similar irrespective of whether depression testing is part of the study design or participants are asked to self-report a diagnosis of depression (12-month prevalence: 8.2% reported by Jacobi et al. [1] tested and 8.1% reported by Thom et al. [34] self-reported).

#### Healthy control group

People without depression are participants from the total population who reported that they were not affected by a depressive disorder (nondepressed German population).

#### Loneliness

The 11-item De Jong Gierveld Loneliness Scale [35] in a German translation was used to assess the extent to which people feel lonely^1^.

#### Number of social relationships

The number of people’s social relationships was measured by the ALLBUS variable on social contacts (V681) [36]. The item asks for the average number of personal daily contacts with other people on a normal weekday. The response categories are 0–4 contacts, 5–9 contacts, 10–19 contacts, 20–49 contacts and more than 50 contacts per day.

### Statistical analyses

#### Hypothesis 1

Spearman’s ρ was chosen as the correlation coefficient, and 95% confidence intervals were calculated after Fieller, Hartley and Pearson [37]. Differences in correlations were tested for significance via Z-standardisation according to Hays [38], with a correction in the calculation of the standard error for Spearman correlation [37].

The results were assessed on the basis of effect size categories proposed by Cohen [39]. For Spearman’s ρ they are: small |ρ| = 0.10, moderate |ρ| = 0.30 and high |ρ| = 0.50.

#### Hypothesis 2

T-test calculation was used to test for overall group differences in feelings of loneliness. Subgroup analyses according to the current phase of depression were performed via univariate analysis of variance with additional contrasts. Welch-ANOVA was chosen as the appropriate procedure for our data, which presented heterogeneity of variance.

The results were assessed based on effect size categories proposed by Cohen [39]. For Cohen’s d they are: > 0,2 small, > 0,5 moderate and > 0,8 high.

#### Hypothesis 3

A Mann-Whitney-U-test was used to test for overall group differences in number of social relationships. Subgroup analyses according to the current phase of depression were performed via a Kruskal-Wallis test and additional Mann-Whitney-U-tests. The effect size *r* of the Mann-Whitney-U-tests as calculated according to Field [40]. It was interpreted also as proposed by Cohen [39]: small *r* =.10, moderate *r* =.30 and high *r* =.50.

### Type of statistical analysis

Statistical analysis was performed after data were collected. The analysis was performed via IBM SPSS Statistics for Windows, version 29.0.2.0.

Power calculation was not performed because of the representative sample, lack of experimental setting, and type of statistical calculations.

For the subgroup analyses of two significance tests in hypothesis 2 and 3, Bonferroni correction was applied [41], leading to a slightly decreased significance level.

## Results

### Characteristics of the participants

For the statistical analyses, the sample of *N* = 5196 participants was adjusted.

#### Exclusion of participants

A total of 1012 people who reported that they thought they had depression but had not been diagnosed by a doctor were excluded from the analyses because of lack of validation of their mental health status.

A relevant variable for the analyses of research questions 2 and 3 was the depression phase. People who stated that they did not know in which phase (acute depressive phase, residual symptomatic phase or not symptomatic phase) of depression they are in (*n* = 24) were excluded due to missing relevant information. People who stated that they did not know how many social contacts they have (*n* = 122) were also excluded, as this information is highly relevant to our study. As 4 people stated both the uncertain phase of depression and the uncertain number of social contacts, the exclusion resulted in an overall sample size of *n* = 4042.

#### Description of the sample

**Table 1:** Descriptives of the sample analysed by main groups

	Sex				Age [years]				
	male	female	diverse		18–29	30–39	40–49	50–59	60–69
Healthy control group (2821)	52.9% (1493)	46.9% (1322)	0.2% (6)		18.3% (515)	18.8% (529)	18.6% (524)	22.5% (635)	21.9% (617)
Depression group (1221)	41% (501)	58.6% (716)	0.3% (4)		16.4% (200)	16.7% (204)	18.1% (220)	27.3% (333)	21.6% (263)

Percentage distribution and absolute numbers (n in brackets) of the sample analysed by sex and age group

In the final sample (*n* = 4042), the people are evenly distributed among the following age groups: 17.7% (*n* = 715) aged 18–29 years, 18.1% (*n* = 733) aged 30–39 years, 18.4% (*n* = 745) aged 40–49 years, 24% (*n* = 968) aged 50–59 years and 21.8% (*n* = 880) aged 60–69 years. The sample consists of 49.3% (*n* = 1994) males, 50.4% (*n* = 2038) females and 0.2% (*n* = 10) diverse individuals. The descriptive distribution of the two main study groups is shown in Table 1.

Among our sample, 30,2% (*n* = 1221) reported having a diagnosis of depression (lifetime prevalence) of whom 22,3% (*n* = 272) reported being in the acute depressive phase, 42,8% (*n* = 523) reported being in the residual symptomatic phase and 34,8% (*n* = 425) reported being in the not symptomatic phase.

### Association between feelings of loneliness and the number of social relationships

#### Descriptives and correlational association

As shown in Table 2, the mean level of loneliness was highest in the acute depressive phase subgroup; the lowest number of social contacts was also found in this subgroup.

In the overall depression group, feelings of loneliness and the number of social relationships were negatively correlated and had descriptively the highest effect size. In the acute depressive subgroup and the residual symptomatic subgroup, significant negative correlations were found, and Spearman’s ρ of the not symptomatic subgroup was not significant. In the healthy control group, a negative correlation was also found, with a smaller effect size. Except for the not symptomatic phase subgroup, all correlations found were significant and similar in direction, and the effect sizes varied but were overall weak in effect size [39].

**Table 2:** Main descriptive findings and correlations

		Level of loneliness	Number of social relationships					Correlation between feelings of loneliness and the number of social relationships	
			0–4	5–9	10–19	20–49	> 50	Spearman’s ρ [95% CI]	*p*
Healthy control group (2821)		M = 4.3 (*SD* = 3.4)	20.5% (577)	29.0% (819)	27.2% (768)	16.4% (462)	6.9% (195)	− 0.132 [-0.169; − 0.094]	< 0.001
Depression group (1221)		M = 6.5 (*SD* = 3.5)	39.2% (479)	31.4% (384)	18.6% (227)	7.9% (96)	2.9% (35)	− 0.234 [-0.288; − 0.179]	< 0.001
Depression subgroups	ADP (272)	M = 8.13 (*SD* = 2.70)	58.5% (159)	23.5% (64)	11.4% (31)	5.1% (14)	1.5% (4)	− 0.176 [-0.292; − 0.054]	< 0.01
	RSP (523)	M = 7.15, (*SD* = 3.24)	40.5% (212)	32.5% (170)	17.4% (91)	7.1% (37)	2.5% (13)	− 0.209 [-0.292; − 0.123]	< 0.001
	NSP (425)	M = 4.74, (*SD* = 3.40)	25.2% (107)	35.3% (150)	24.7% (105)	10.6% (45)	4.2% (18)	− 0.092 [-0.188; 0.006]	. 059

Percentage distributions and absolute numbers (n in brackets). ADP = acute depressive phase, RSP=residual symptomatic phase, NSP = not symptomatic phase

#### Association differences tested for significance

See Table 3 for all the applied comparisons and statistical parameters.

Overall, less feelings of loneliness were associated with a higher number of social relationships, with a significantly greater effect size in the depression group compared with the healthy control group.

**Table 3:** Differences in correlations compared and tested for significance

		Spearman’s ρ of healthy control group (-0.132 [-0.169; − 0.094])	Spearman’s ρ of depression subgroup RSP (-0.209 [-0.292; − 0.123])	Spearman’s ρ of depression subgroup NSP (-0.092 [-0.188; 0.006])
		Z (*p*)	Z (*p*)	Z (*p*)
Spearman’s ρ of depression group (-0.234 [-0.288; − 0.179])		2.99 (< 0.003)		
Spearman’s ρ of depression subgroups	ADP (-0.176 [-0.292; − 0.054])	0.69 (0.49)	0.44 (0.66)	1.09 (0.29)
	RSP (-0.209 [-0.292; − 0.123])	1.61 (0.11)	-	1.78 (0.08)
	NSP (-0.092 [-0.188; 0.006])	0.75 (0.45)	1.78 (0.08)	-

Spearman’s ρ correlations compared via Z-standardisation according to Hays [38], with corrections after Fieller, Hartley and Pearson [37]. ADP = acute depressive phase, RSP=residual symptomatic phase, NSP = not symptomatic phase

In the subgroups of people with depression, the correlations must be accepted as similar in effect size as in the healthy control group, and no statistical significance was shown. The two significant correlations of the acute and residual symptomatic depressive phases were also not significantly different from those of any other subgroup. Additionally, all correlational differences between the not symptomatic depressive phase subgroup and the other subgroups, such as the correlation of the NSP subgroup itself, were not statistically significant (Table 3).

The results are contrary to the expectations, and hypothesis 1 must be rejected in two respects.

First, the association between feelings of loneliness and the number of social relationships were similar in direction in all groups and were significantly greatest in the overall depression group.

Second, subgroup analyses according to the depressive phase revealed no significant effects.

### Feelings of loneliness in the depression group

The statistical calculation revealed a moderate and significant effect of depression on feelings of loneliness. Overall, the group comparisons revealed that people with depression (*M* = 6.53, *SD* = 3.45) had significantly more feelings of loneliness compared with the healthy control group (*M* = 4.30, *SD* = 3.38), *t*(4040) = 19.08, *p* <.001 (one-sided), d = 0.654.

Additional univariate analysis of variance (Welch-ANOVA) revealed significant differences according to the current phase of depression, Welch’s *F*(2, 729.46) = 114.34, *p* <.001.

Detailed post-hoc analyses with all subgroup differences calculated are presented in Supplementary Table 1 [see Additional File 1], the overall subgroup analyses revealed significant differences between all groups.

The depression subgroup differences addressed in hypothesis 2 were tested by calculating the following two contrasts as part of the Welch-ANOVA:

1. Contrast of ADP versus RSP and NSP combined
2. U-test of RSP versus NSP

The calculation of the first contrast revealed that, statistically, the level of feelings of loneliness was significantly greater in the acute depressive subgroup (M = 8.13, *SD* = 2.70) than in the residual symptomatic subgroup (M = 7.15, *SD* = 3.24) and in the not symptomatic subgroup (M = 4.74, *SD* = 3.38) combined, with a mean difference of 2.18 (*SE* = 0.22), *p* <.002, d = 0.69; this corresponds to a moderate effect. The outcome of the second contrast calculation was that there was a statistically significant difference in the residual symptomatic subgroup (M = 7.15, *SD* = 3.24) compared with the not symptomatic subgroup (M = 4.74, *SD* = 3.40) of 2.40 (*SE* = 0.21), *p* <.002, d = 0.73, which corresponds to a moderate effect also.

Overall, both contrasts in feelings of loneliness in the depression subgroups were significant and moderately high in effect size [39].

The results were in line with the expectations and hypothesis 2 was confirmed.

### Number of social relationships in the depression group

The statistical calculation revealed a small significant effect of depression on the number of social relationships. Descriptively, people with depression had fewer social relationships than the healthy control group (Table 2). When the two groups of interest were statistically examined, the preconditions were met, Kolmogorov-Smirnov *p* <.001. There was a statistically significant difference in the number of social relationships between people with depression and the healthy control group, *U* = 1247054,50, *Z* = -14.44, *p* <.001, *r* = .227.

Additionally, the Kruskal-Wallis test revealed significant differences according to the current phase of depression, *H*(2) = 77.131, *p* <.001.

Detailed post-hoc analyses with all subgroup differences calculated are presented in Supplementary Table 2 [see Additional File 2], the overall subgroup analyses revealed significant differences between all groups.

The depression subgroup differences addressed in hypothesis 3 were tested by calculating the following two tests:

1. U-test of ADP versus RSP and NSP combined.
2. U-test of RSP versus NSP.

The calculation of the first combined U-test revealed that the number of social relationships differed significantly between the acute depressive subgroup and both other subgroups, *U* = 94491,000, *Z* = -7.080, *p* <.002, *r* = .203. The outcome of the second test calculation was a statistically significant difference in the residual symptomatic subgroup compared with the not symptomatic subgroup, *U* = 90208,500, *Z* = -5.221, *p* <.002, *r* = .170.

Overall, both differences in the number of social relationships depending on the current phase of depression were significant, but the effect sizes small [39]. 

The results were in line with the expectations and hypothesis 3 was confirmed.

## Discussion

### Main research question

The main aim of this study was to explore the association between the subjective and objective domains of social relationships in people with depression. The main finding was contrary to our expectations, feelings of loneliness (subjective domain) and the number of social relationships (objective domain) were more strongly negatively associated in people with depression (overall lifetime prevalence) than in healthy people. Additionally, we did not find significant state-dependent effects regarding the differences in three depression subgroups.

While more feelings of loneliness are correlated with fewer social relationships in healthy people [29], the fact that feelings of loneliness can be part of depressive symptomatology [7] and previous findings in PDD patients [29] led to our initial hypothesis, that there would be no correlation in people with depression.

Our empirical research now indicates that people with depression have more feelings of loneliness when they have fewer social relationships. It seems that research on people with PDD cannot be used to justify assumptions about people with depression regarding this association. This may be due to differences in the characteristics of PDD and episodic depression patients, and our study may be seen as a starting point for further research on differences in social relationship domains across depressive disorders.

An explanation for our findings may be our methodological choice to focus on two specific relationship domains. In doing so, we addressed the current lack of clarity in the field of loneliness research (subjective and objective) [14, 25]. However, we are consequently not in possession of research that is directly comparable. Our findings establish a starting point for further research on specific social relationship domains and their interactions in people with depression.

The effect sizes of the examined correlations are also contrary to our expectations. The lower effect size in the healthy control group may be explained by the multidimensional nature of social relationship domains [25], of which this study focused on two specific domains. The significantly greater effect size in the overall depression group may be due to greater variance in feelings of loneliness and in the number of social relationships across the different phases of depression that were combined in this group. A greater variance leads to higher effect sizes. This is a consequence of the statistical nature of the correlations. A detailed examination of the correlations in the depression subgroups revealed that people in an acute depressive phase or a residual symptomatic phase presented similar correlations, albeit not as high as those in the overall depression group. In the present study, people in a not symptomatic phase of depression seemed to be involved in the subjective and objective domains of social relationships more independently.

The differences in correlations can mainly be explained by differences in feelings of loneliness, as the calculations for hypothesis 2 show. Feelings of loneliness are likely to depend more on the current state of depression, since they are associated with depressive symptoms [6]. Also, feelings of loneliness presumably influence the subjective perception of social relationships. The correlational differences observed between the overall depression group and the subgroups may be explained by greater variance in feelings of loneliness due to these two aspects. This is discussed in more detail in the secondary research questions section.

The implication of the association found between the subjective and objective social relationship domains in people with depression is that both domains need to be addressed in the treatment of depression. We provide empirical support for clinicians who are already addressing the different domains in their treatment. Our results also suggest that merely assessing the number of one’s social relationships is associated with feelings of loneliness in people with a lifetime diagnosis of depression. This may have implications for depression interventions and forms of (social) support, namely involving objective social relationships could be helpful.

### Secondary research questions

This study aimed to explore two well-known effects on the basis of single-source data from a population survey in Germany: first, depression is related to higher levels of loneliness, and second, depression is associated with fewer social relationships. The results revealed significant effects for both research subjects, in accordance with the hypotheses.

The first effect was expected, because of previous findings of a moderate correlation between feelings of loneliness and depression, and the understanding that feelings of loneliness are part of the symptomatology and diagnosis of depression [4–7].

The second effect was presumed, because of previous findings of social withdrawal in depression and reduced interest in social interaction in people with depression [20–22].

Our results therefore indicate a replication of findings of both effects. These are based on a recent representative national dataset. The insight into the simultaneous existence of both effects is a strength of our study.

Additional subgroup analyses revealed that the effect of the depression phase group on feelings of loneliness was moderately high, whereas the effect on the number of social relationships was small.

The use of single-source data makes it possible to assume that feelings of loneliness vary more with the current state of episodic depression than the number of social relationships. An explanation for this finding may be that subjective feelings are more susceptible to phases with depressive symptoms because they can be an integral part of them, and because depression is an affective disorder in general. On the other hand, actual social relationships may be a more stable feature of people’s environment across different states of depression.

The treatment of acute depression may be impacted by the finding of greater variation in feelings of loneliness depending on the current state of depression. This finding suggests that, when treating acute depression, it may be beneficial to focus on the subjective perception of social relationships before considering approaches to improve more objective domains of social relationships.

Both social relationship domains affect people with depression. Because depression is such a highly prevalent psychiatric disorder [1, 2], it is of particular interest to clinicians, patients, relatives and policymakers to understand it more fully. This requires continuous updating of the data, which we provide here.

### Limitations

An important limitation of our study is our reliance on self-report measures. Self-reported depression diagnoses are likely to be less reliable than clinical assessments are. Furthermore, similar outcomes regarding the prevalence of depression, as determined through self-reports [34] or clinical interviews [1], do not guarantee that affected individuals are correctly identified. The same applies to the measurement of the current state of episodic depression. Further research could use a more objective approach to measure the current state of depression rather than relying solely on participants’ self-reports.

In line with this limitation, the ‘healthy control group’ is potentially heterogeneous. While this group is clearly defined as individuals without a self-reported depression diagnosis, it is possible that people with other depressive disorders may contaminate this research group. Although online self-reporting is an efficient and widely used method for collecting representative population data, it is important to consider these limitations.

There is recent evidence that the 11-item De Jong Gierveld Loneliness Scale lacks high content and construct validity [42]. However, the scale is currently still widely used because its structural validity and internal consistency are acceptable. For future research, careful consideration should be given to the use of this scale.

The 11-item De Jong Gierveld Loneliness Scale consists of two subscales that were not considered separately in this study, subsequent research could examine the relationships between depression and emotional and social loneliness to improve our understanding of these.

Asking people with acute depressive symptoms about their ‘average number of personal daily contacts with other people on a normal weekday’ may distort the results regarding the number of social relationships, because the ‘normal’ weekday structure is lost during an acute phase of illness. Further research could specify what is meant by ‘normal’ when this item is used.

Finally, the lack of control over confounding variables such as age, sex, or other severe disorders must be acknowledged as a limitation of the study. Further research in this field may be able to address this deficiency.

## Conclusion

By examining two specific social relationship domains (subjective and objective), our research indicates a negative correlation between feelings of loneliness and the number of social relationships among people with depression. This inverse association between the two domains has implications for treatment decisions and the content of intervention programs.

Additionally, our recent representative single-source data from the German population indicate that people with depression exhibit increased levels in both domains. With respect to the current state of depression, we find greater variation in feelings of loneliness than in the number of social relationships. One important potential implication is the idea of addressing both social relationship domains differently in therapy, with an understanding of their dynamics across the different states of depression.

Overall, these findings improve our understanding of the role of social relationships in depression. 

## Supplementary Information

Below is the link to the electronic supplementary material.

Supplementary Material 1

Supplementary Material 2

## Data Availability

The datasets used and analysed during the current study are available from the corresponding author on reasonable request.

## Footnotes

1 For a critical review of the scale, see Alsubheen et al. [42].
