# Supplementary material for: The association between feelings of loneliness and the number of social relationships in depression: a cross-sectional study of German adults
Source: BMC Psychiatry. 2026 Feb 28;26:226. doi: 10.1186/s12888-026-07915-3 (PMC12964965; doi:10.1186/s12888-026-07915-3)
Supplement: Supplementary file 1 — Supplementary Material 1 [file 12888_2026_7915_MOESM1_ESM.docx]

**Supplementary Table 1.** Multiple comparisons of feelings of loneliness between all depression subgroups.

| Phase of depresion (I) | Phase of depression (J) | Mean Difference (I-J) | Std. Error | *p* | 95% Confidence Interval |
| --- | --- | --- | --- | --- | --- |
| ADP | RSP | .98 | 0.24 | <.001 | [.41; 1.55] |
|  | NSP | 3.38 | 0.25 | <.001 | [2.79; 3.98] |
| RSP | ADP | -.98 | 0.24 | <.001 | [-1.55; -.41] |
|  | NSP | 2.40 | 0.21 | <.001 | [1.91; 2.90] |
| NSP | ADP | -3.38 | 0.25 | <.001 | [-3.98; 2.79] |
|  | RSP | -2.40 | 0.21 | <.001 | [-2.90; -1.91] |

Welch-ANOVA post-hoc subgroup comparisons after Bonferroni, with *p* for multiple tests. ADP = acute depressive phase, RSP=residual symptomatic phase, NSP=not symptomatic phase.
