# Supplementary material for: The association between feelings of loneliness and the number of social relationships in depression: a cross-sectional study of German adults
Source: BMC Psychiatry. 2026 Feb 28;26:226. doi: 10.1186/s12888-026-07915-3 (PMC12964965; doi:10.1186/s12888-026-07915-3)
Supplement: Supplementary file 2 — Supplementary Material 2 [file 12888_2026_7915_MOESM2_ESM.docx]

**Supplementary Table 2.** Multiple comparisons of the number of social relationships between all depression subgroups.

| Subgroup 1-Subgroup 2 | Test Statistic | Std. Error | Std. Test Statistic | *p* |
| --- | --- | --- | --- | --- |
| ADP-RSP | -112.03 | 25.01 | -4.48 | <.001 |
| ADP-NSP | -225.57 | 25.98 | -8.68 | <.001 |
| RSP-NSP | -113.55 | 21.85 | -5.20 | <.001 |

Kruskal-Wallis post-hoc subgroup comparisons after Bonferroni. with *p* for multiple tests. Each row tests the null hypothesis that the subgroup 1 and subgroup 2 distributions are the same. Asymptotic significances (2-sided tests) are displayed. ADP = acute depressive phase, RSP=residual symptomatic phase, NSP=not symptomatic phase.
